# Association of Small Dense LDL Serum Levels and Circulating Monocyte Subsets in Stable Coronary Artery Disease

**DOI:** 10.1371/journal.pone.0123367

**Published:** 2015-04-07

**Authors:** Konstantin A. Krychtiuk, Stefan P. Kastl, Stefan Pfaffenberger, Max Lenz, Sebastian L. Hofbauer, Anna Wonnerth, Lorenz Koller, Katharina M. Katsaros, Thomas Pongratz, Georg Goliasch, Alexander Niessner, Ludovit Gaspar, Kurt Huber, Gerald Maurer, Elisabeth Dostal, Johann Wojta, Stanislav Oravec, Walter S. Speidl

**Affiliations:** 1 Department of Internal Medicine II—Division of Cardiology, Medical University of Vienna, Waehringerguertel 18–20, 1090, Vienna, Austria; 2 Ludwig Boltzmann Cluster for Cardiovascular Research, Waehringerguertel 18–20, 1090, Vienna, Austria; 3 Krankenanstalten Dr. Dostal, Saarplatz 9, 1190, Vienna, Austria; 4 2nd Department of Internal Medicine, Faculty of Medicine, Comenius University, Vajanského nábrežie, 811 02, Bratislava, Slovakia; 5 3rd Medical Department, Wilhelminenhospital, Montleartstraße 37, 1160, Vienna, Austria; 6 Core Facilities, Medical University of Vienna, Waehringerguertel 18–20, 1090, Vienna, Austria; University Heart Center Freiburg, GERMANY

## Abstract

**Objective:**

Atherosclerosis is considered to be an inflammatory disease in which monocytes and monocyte-derived macrophages play a key role. Circulating monocytes can be divided into three distinct subtypes, namely in classical monocytes (CM; CD14++CD16-), intermediate monocytes (IM; CD14++CD16+) and non-classical monocytes (NCM; CD14+CD16++). Low density lipoprotein particles are heterogeneous in size and density, with small, dense LDL (sdLDL) crucially implicated in atherogenesis. The aim of this study was to examine whether monocyte subsets are associated with sdLDL serum levels.

**Methods:**

We included 90 patients with angiographically documented stable coronary artery disease and determined monocyte subtypes by flow cytometry. sdLDL was measured by an electrophoresis method on polyacrylamide gel.

**Results:**

Patients with sdLDL levels in the highest tertile (sdLDL≥4mg/dL;T3) showed the highest levels of pro-inflammatory NCM (15.2±7% vs. 11.4±6% and 10.9±4%, respectively; p<0.01) when compared with patients in the middle (sdLDL=2-3mg/dL;T2) and lowest tertile (sdLDL=0-1mg/dL;T1). Furthermore, patients in the highest sdLDL tertile showed lower CM levels than patients in the middle and lowest tertile (79.2±8% vs. 83.9±7% and 82.7±5%; p<0.01 for T3 vs. T2+T1). Levels of IM were not related to sdLDL levels (5.6±4% vs. 4.6±3% vs. 6.4±3% for T3, T2 and T1, respectively). In contrast to monocyte subset distribution, levels of circulating pro- and anti-inflammatory markers were not associated with sdLDL levels.

**Conclusion:**

The atherogenic lipoprotein fraction sdLDL is associated with an increase of NCM and a decrease of CM. This could be a new link between lipid metabolism dysregulation, innate immunity and atherosclerosis.

## Background

Atherosclerosis is considered to be an inflammatory process in which monocytes and monocyte-derived macrophages play a key role in both initiation and progression of the disease.[1, 2] Circulating monocytes can be divided into three distinct subtypes according to their surface expression of CD14 and CD16.[3, 4] Classical monocytes (CM; CD14++CD16-) account for approximately 90% of all circulating monocytes. CD16-positive monocytes namely intermediate monocytes (IM; CD14++CD16+) and non-classical monocytes (NCM; CD14+CD16++) show a pro-inflammatory phenotype, exhibit an increased production of inflammatory cytokines upon stimulation and are elevated in chronic inflammatory diseases.[5–7]

Furthermore, the CD16+ monocyte population was shown to be expanded in patients suffering from stable coronary artery disease (CAD) and correlated with intima-media thickness and BMI in apparently healthy adults.[8, 9] The proportion of NCM was strongly elevated in obese patients, correlated with fasting glucose and fat mass and decreased together with intima-media thickness during weight loss.[10] Additionally, an inverse correlation between NCM and HDL-cholesterol has been demonstrated, while total cholesterol and triglycerides were positively correlated with NCM.[11, 12] In a study involving more than 900 patients undergoing elective coronary angiography, the proportion of IM predicted future cardiovascular events.[13]

Elevated total cholesterol and low density lipoprotein (LDL)-cholesterol levels have long been identified as potent risk factors in atherogenesis.[14, 15] However, low-density lipoproteins are a heterogeneous class of particles and accumulating evidence suggests that different LDL subfractions vary in their risk profile.[15–17] Thus, patients with the same LDL-levels may be at different cardiovascular risk. Indeed, small dense LDL (sdLDL) represent an emerging cardiovascular risk factor, independent of traditional risk factors including total LDL levels.[18–20] Several studies implicated a direct role of sdLDL in atherogenesis and thus provided evidence that the role of sdLDL goes beyond a simple marker of metabolic disturbances. These particles exhibit reduced binding capacities to LDL-receptors and show a stronger affinity to the extracellular matrix within the vascular wall making them more prone to oxidative modification.[16, 21]

The mechanism leading to elevated levels of inflammatory monocyte subpopulations in patients with atherosclerotic vascular disease is poorly understood. Therefore, the aim of this study was to examine whether monocyte subsets are associated with sdLDL in patients with stable, coronary artery disease. In addition, we tested whether sdLDL serum levels correlate with pro- and anti-inflammatory cytokines.

## Materials And Methods

### Subjects and study design

This is a single-center, cross-sectional study. Between September 2009 and April 2010, we recruited ninety consecutive patients with stable CAD undergoing elective coronary angiography. Patients gave written, informed consent for this study, which was approved by the ethical committee of the Medical University of Vienna and complies with the Declaration of Helsinki. Inclusion criteria comprised male and female patients aged > 18 years with stable CAD undergoing elective coronary angiography. Exclusion criteria consisted of recent acute coronary syndrome, defined as ST-elevating myocardial infarction (STEMI), non-STEMI or unstable angina with or without percutaneous coronary intervention (PCI) within the last three months, heart failure, valvular disease, malignant disease, liver, kidney or other acute or chronic inflammatory diseases. Arterial hypertension was defined as systolic blood pressure ≥ 140 mmHg, diastolic blood pressure ≥ 90 mmHg in at least two measurements or the current use of antihypertensive drugs. Subjects were defined as being diabetic if treated for insulin or non-insulin-dependent diabetes mellitus or plasma fasting glucose ≥ 126 mg/dL in at least two measurements. Extent of coronary artery disease is given as the number of epicardial coronary arteries with a ≥70% stenosis. High-dose statin treatment was defined as treatment with atorvastatin with a dosage of at least 40mg or rosuvastatin at a dosage of at least 10mg daily.

### Blood sampling

Blood was drawn in the morning prior to elective coronary angiography after venipuncture from an antecubital vein using a 21-gauge butterfly needle (0.8 mm × 19 mm; Greiner Bio-One, Kremsmünster, Austria). After the initial 3 mL of blood were discarded, blood was drawn into an EDTA tube (Greiner Bio-One) for immediate analysis by flow cytometry. Furthermore, a 3.8% sodium citrate Vacuette tube (Greiner Bio-One; nine parts of whole blood, one part of sodium citrate 0.129 M/L), a serum separator tube (Greiner Bio-One) and an EDTA tube (Greiner Bio-One) were collected, immediately centrifuged (4°C; 3000RPM for 15 min) and stored at—80°C for later analysis.

### Laboratory measurements

Standard laboratory measurements including high-sensitive C-reactive protein (hsCRP) were analyzed in the central laboratory of the General Hospital of Vienna. For the measurement of plasma interleukin-6 (IL-6), a specific enzyme-linked immunosorbent assay (ELISA) was used (Human IL-6 Quantikine high-sensitivity ELISA Kit, R&D Systems, Minneapolis, MN, USA, catalog number HS600B). Plasma levels of interleukin-10 (IL-10) and tumor necrosis factor-α (TNF-α) were determined using a customized multiplex assay (Luminex Assay, R&D Systems, catalog number FCST03) according to manufacturer's instructions. In 37% of patients, TNF-α levels were undetectable and thus were set to 15 pg/mL, representing the lowest detection limit.

### Flow cytometry

**Fig 1:**
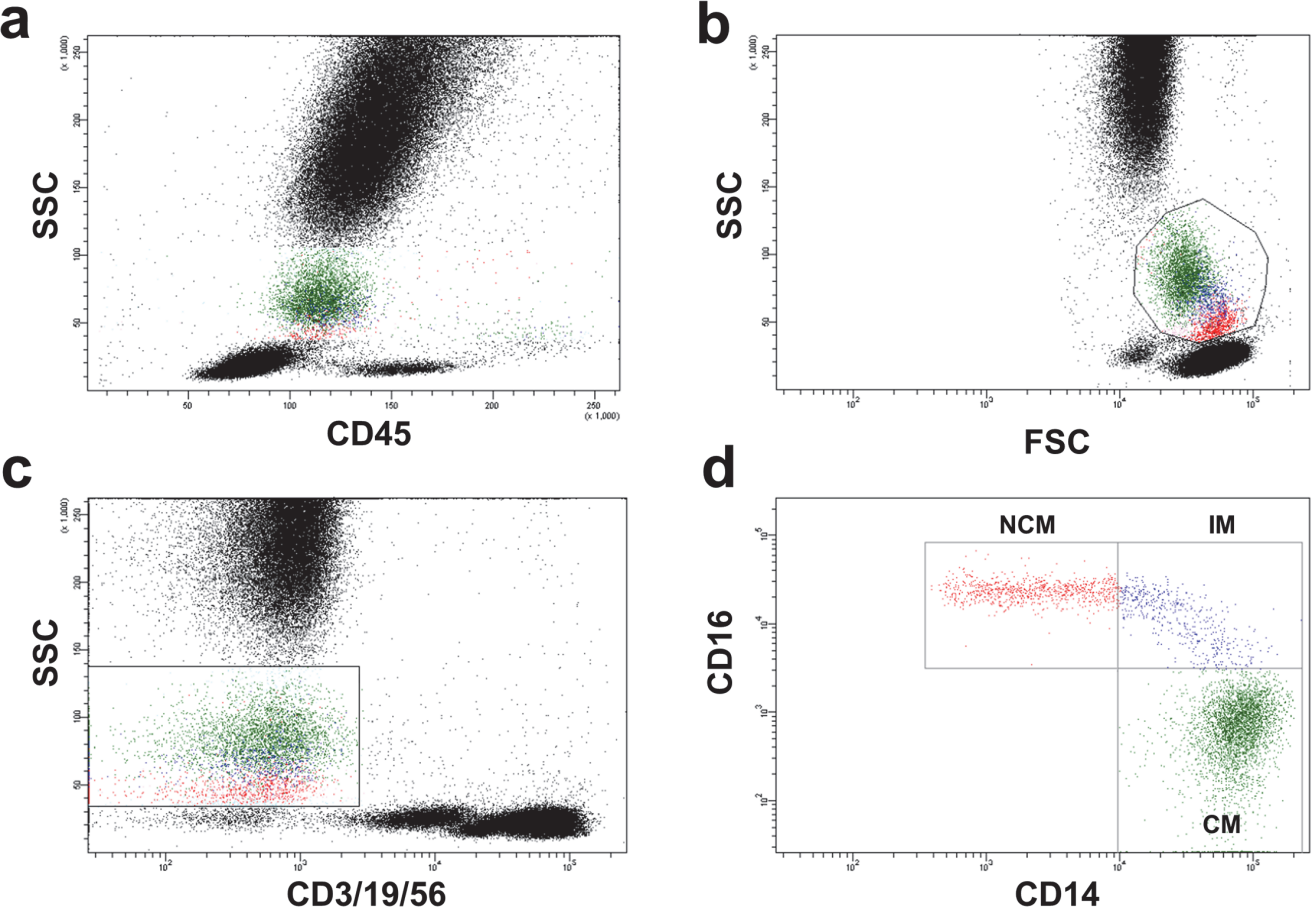
Gating strategy used for monocyte subset discrimination. Monocytes were defined as CD45 positive cells (B) exhibiting a typical forward (FSC) and sideward scatter (SSC) profile (A). To exclude possible contamination with T-cells, B-cells and Natural Killer cells, cells that stained for CD3, CD19 and CD56 were excluded, respectively (C). Remaining CD45+CD3/19/56- cells with a typical FSC/SSC profile were considered monocytes and distinguished according to their CD14 and CD16 surface expression into classical monocytes (CD14++CD16-), intermediate monocytes (CD14++CD16+) and non-classical monocytes (CD14+CD16++) (D).

Whole blood flow cytometry for determination of leukocyte and monocyte subset distribution was performed using a FACS Canto II with the FACS Diva Software (both Becton Dickinson). The staining and gating strategy is outlined in Fig 1. Briefly, 100 μL of EDTA-anticoagulated whole blood was stained with saturating concentrations of the following fluorochrome-conjugated monoclonal antibodies (mAbs): Peridinin chlorophyll protein (PerCP)-labeled mAb for CD45 (Beckton Dickinson, catalog number 345809), fluorescein isothiocyanate (FITC)-labeled mAb for CD14 (Beckton Dickinson, catalog number 345784), allophycocyanin (APC)-H7-labeled mAb for CD16 (Beckton Dickinson, catalog number 560195), APC- labeled mAb for CD3 (Beckton Dickinson, catalog number 345767), CD19 (Beckton Dickinson, catalog number 345791) and CD56 (Beckton Dickinson, catalog number 341027) and corresponding isotype controls. After incubation for 15 minutes in the dark, 1.5 mL lysing solution (BD FACS lysing solution BD Biosciences) was added. After an additional 15 minutes of incubation in darkness, cells were washed three times by adding 1mL PBS and centrifugation at 820 RPM for 5 minutes each. Cells were then resuspended in 1mL fixative solution (FACS Flow, reagent-grade water and BD Cellfix) for FACS-analysis. Monocytes were identified as CD45-positive and CD3-, CD19- and CD56-negative cells exhibiting a specific forward and sideward scatter profile. Individual monocyte subsets were defined according to a recently published international consensus document as "classical monocytes" (CM; CD14++CD16-), "intermediate monocytes" (IM; CD14++CD16+) and "non-classical monocytes" (NCM; CD14+CD16++).[4]

### Lipid measurements

**Fig 2:**
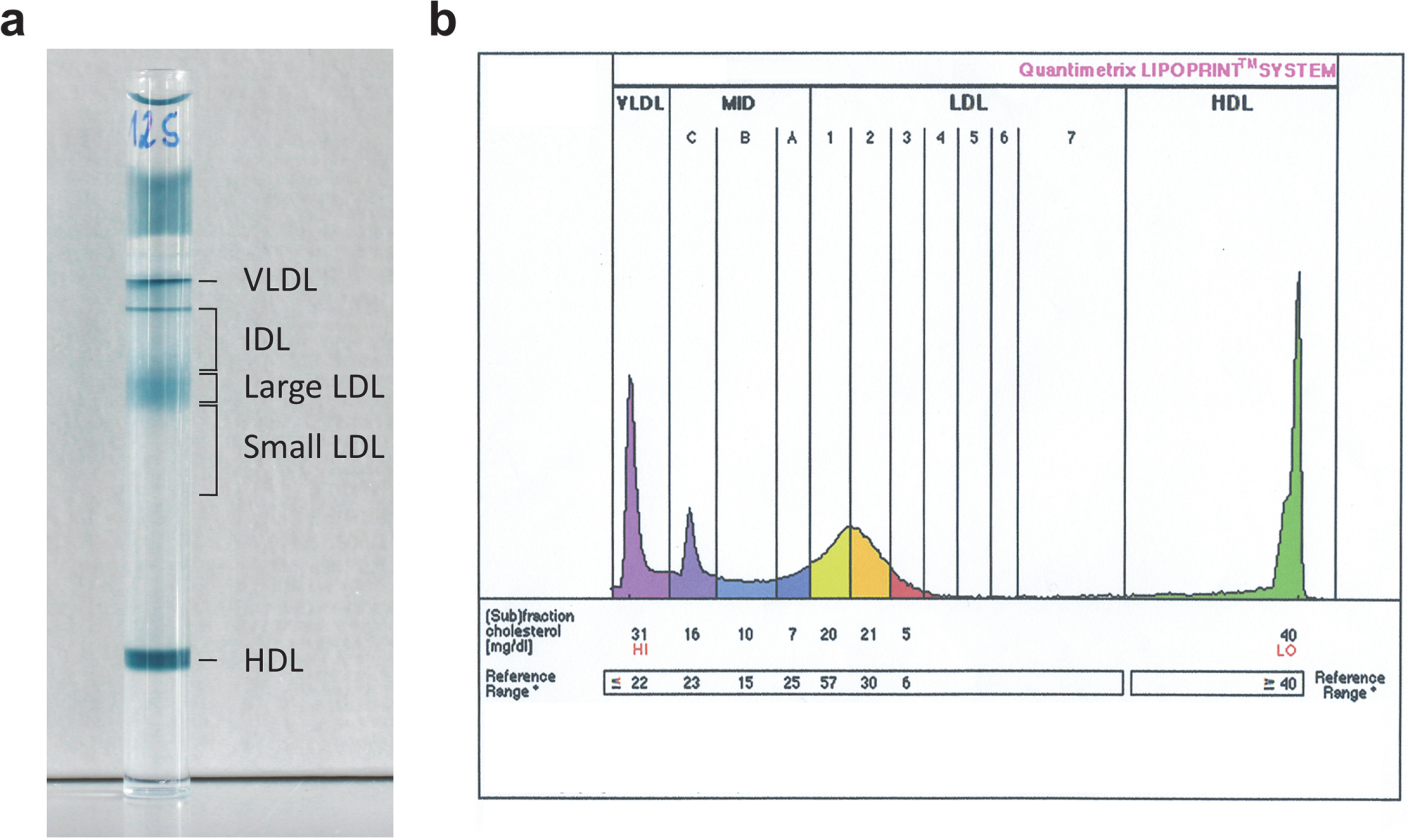
Representative image of a polyacrylamide gel electrophoresis tube and a corresponding analysis as obtained from the Lipoprint system. a) polyacrylamide gel electrophoresis tube b) MID A-C represent IDL, LDL subfractions 1 and 2 represent large LDL and LDL subfractions 3–7 represent small, dense LDL

For all lipid measurements, serum samples were used immediately after thawing from -80°C. Levels of total cholesterol, HDL, LDL and triglycerides were measured by the general laboratory of *Krankenanstalten Dr*. *Dostal* using enzymatic methods. For lipid subfraction quantification, the Quantimetrix LDL Lipoprint System (Quantimetrix Corporation, Redondo Beach, CA, USA), was used according to manufacturer's instructions as described elsewhere.[22, 23] In brief, this method is based on high resolution polyacrilamide gel electrophoresis, separating and measuring total VLDL and subfractions of LDL and IDL. LDL were divided into seven subfractions, with subfraction 1 representing large LDL particles, subfraction 2 constituting intermediate LDL particles, while subfractions 3–7 account for small dense LDL particles (see Fig 2 for a representative image of a polyacrylamide gel electrophoresis tube and the corresponding analysis). IDL were divided into large, medium and small IDL subfractions. LDL particle size was calculated as previously described.[22]

### Statistical analysis

Categorical variables are expressed as counts or percentages and were compared by the χ^2^ or by Fisher’s exact test where appropriate. Continuous variables are given as mean ± standard deviation or as median (interquartile range). Parametric data was compared using the unpaired Student's t-test or by ANOVA, while skewed data (assessed by the Kolmogorov–Smirnov test) was compared by the unpaired Student's t-test or by ANOVA after log-transformation. Correlations were calculated using Pearson’s correlation coefficient. A value of p<0.05 (two-tailed) was considered statistically significant. All statistical analyses were performed with the Predictive Analysis SoftWare PASW Statistics 18.0 (IBM, Armonk, NY, USA).

## Results

### Patient characteristics

We included 90 patients with angiographically proven stable coronary artery disease. Clinical characteristics of the patient population are given in Table 1. 80% of patients were male, 89% of patients were hypertensive, 30% of patients had diabetes and 23% were smokers.

**Table 1:** Clinical characteristics of the study population.

	**Total**	**sdLDL tertile 1**	**sdLDL tertile 2**	**sdLDL tertile 3**	**p-value**
	**n = 90**	**n = 35**	**n = 30**	**n = 25**	
**sdLDL (mg/dL)**	0–24	0–1	2–3	4–24	
**Age (years)**	64.1 ± 1.0	66.1 ± 10.6	63.7 ± 9.6	61.8 ± 6.8	0.23
**Male gender, n (%)**	72 (80)	25 (71.4)	24 (80)	23 (31.9)	0.15
**Hypertension, n (%)**	80 (88.9)	30 (85.7)	26 (86.7)	24 (96)	0.41
**Diabetes Mellitus, n (%)**	27 (30)	11 (31.4)	7 (23.3)	9 (36)	0.58
**Current smoker, n (%)**	21 (23.3)	10 (28.6)	5 (16.7)	6 (24)	0.53
**CAD Extent (VD)**					0.41
**1VD, n (%)**	25 (27.8)	12 (34.3)	7 (23.3)	6 (24)	
**2VD, n (%)**	36 (40)	16 (45.7)	11 (36.7)	9 (36)	
**3VD, n (%)**	29 (32.2)	7 (20)	12 (40)	10 (40)	
**Statin Treatment**					0.44
**No Statin, n (%)**	15 (16.7)	7 (20)	6 (20)	2 (8)	
**Low-dose Statin, n (%)**	47 (52.2)	16 (45.7)	14 (46.7)	17 (68)	
**High-dose Statin, n (%)**	28 (31.1)	12 (34.3)	10 (33.3)	6 (24)	
**BMI (kg/m** ^**2**^ **)**	29 ± 4.7	29.7 ± 5.5	28.1 ± 5.5	29.2 ± 4.2	0.37
**HbA1c (%)**	6.1 ± 0.9	5.9 ± 0.8	6.3 ± 1	6.2 ± 1	0.29
**Creatinine (mg/dL)**	1.1 ± 0.3	1.1 ± 0.3	1.1 ± 0.2	1.2 ± 0.4	0.14
**Leukocytes (G/L)**	7.1 ± 1.7	6.9 ± 1.6	7 ± 1.7	7.4 ± 1.9	0.57
**Triglycerides (mg/dL)**	153.3 ± 81.2	119 ± 38	144.6 ± 62.7	211.8±110.8	<0.001
**TC (mg/dL)**	164.6 ± 39	148 ± 35.9	163.9 ± 32.4	188.7 ± 39.4	<0.001
**HDL (mg/dL)**	40.1 ± 13.4	39.9 ± 12.4	43.4 ± 14.9	39.3 ± 13	0.46
**VLDL (mg/dL)**	28.6 ± 9.4	25.0 ± 7.6	27.4 ± 7.8	34.9 ± 10.6	<0.001
**LDL (mg/dL)**	93.3 ± 30.8	84.3 ± 31.6	92.3 ± 24.2	107 ± 32.8	0.016
**Mean LDL particle size (nm)**	266.8 ± 2.7	268.9 ± 1.0	266.9 ± 0.9	263.7 ± 2.8	<0.001

sdLDL small dense low density lipoprotein; CAD coronary artery disease; VD vessel disease; BMI body mass index; TC total cholesterol; HDL high density lipoprotein; VLDL very low density lipoprotein; LDL low density lipoprotein;

### Correlation of lipid parameters, statin therapy and disease severity with circulating monocyte subsets

Mean number of CM as determined by flow cytometry (Fig 1) were 270.5±142.7 cells/μL (82.1±6.7% of total monocytes), mean number of circulating NCM were 39.7±28.9 cells/μL (12.3±5.9% of total monocytes) and mean number of IM were 18.7±15.1 cells/μL (5.6±3.3% of total monocytes). Triglyceride levels, very low density lipoprotein (VLDL) and high density lipoprotein (HDL) levels did not correlate with monocyte subset distribution (Table 2). Serum levels of total cholesterol (R = -0.25, p = 0.02) and LDL (R = -0.25, p = 0.017) were inversely correlated with circulating CM. In addition LDL showed also a correlation with IM (R = 0.20, p = 0.05) but not with NCM (R = 0.17, p = 0.10). In contrast, sdLDL correlated with numbers of circulating pro-inflammatory NCM (R = 0.25, p = 0.017) but not with CM (R = -0.2, p = 0.06) or IM (R = -0.04, p = 0.70). In addition, LDL particle size correlated inversely with NCM (R = -0.23, p = 0.03) but not with CM (R = 0.14, p = 0.19) or IM (R = 0.13, p = 0.24). 52.2% of patients received low-dose statin treatment and 31.1% were on a high-dose statin regimen, while 16.7% of patients were not treated with statins. Interestingly, statin treatment and dose was not associated with distribution of monocyte subsets (data not shown). The presence of multivessel disease (i.e. two or more coronary arteries with ≥70% stenosis) was significantly associated with an increased proportion of circulating NCM (10.6±7.0% vs. 13.0±5.2%; p = 0.015). In contrast, circulating CM (84.0±7.2% vs. 81.3±6.4%; p = 0.10) and IM (5.4±2.5% vs. 5.7±3.6%; p = 0.83) were not associated with severity of CAD.

**Table 2:** Correlation of lipid parameters and circulating monocyte subsets.

**Monocyte subsets**	**Classical monocytesCD14++CD16-**		**Intermediate monocytesCD14++CD16+**		**Non-classical monocytesCD14+CD16++**	
	**R**	**p-value**	**R**	**p-value**	**R**	**p-value**
**Triglycerides**	-0.04	0.71	0.036	0.74	0.025	0.82
**Total Cholesterol**	**-0.25**	**0.02**	0.14	0.18	0.20	0.06
**HDL**	-0.08	0.45	-0.06	0.60	0.12	0.25
**VLDL**	-0.13	0.21	0.13	0.22	0.08	0.47
**LDL**	**-0.25**	**0.017**	**0.20**	**0.05**	0.17	0.10
**sdLDL**	-0.20	0.06	-0.04	0.70	**0.25**	**0.017**
**Mean LDL particle size**	0.14	0.19	0.13	0.24	**-0.23**	**0.03**

HDL high density lipoprotein; VLDL very low density lipoprotein; LDL low density lipoprotein; sdLDL small dense low density lipoprotein; significant correlations are printed bold.

### Association of sdLDL and cardiac risk factors

Patients were divided into three groups according to sdLDL tertiles (lowest tertile sdLDL = 0-1mg/dL, second tertile sdLDL = 2-3mg/dL, third tertile sdLDL≥4mg/dL). As outlined in Table 1, there were no differences in terms of presence or absence of traditional risk factors for atherosclerosis such as presence of hypertension, diabetes mellitus or smoking between patients with low or high sdLDL. Interestingly, statin treatment did not differ between patients according to their sdLDL tertiles. However, total cholesterol, LDL, VLDL and triglyceride levels differed between patient groups, with highest levels seen in patients in the highest sdLDL tertile.

### Monocyte subsets are associated with the highest tertile of sdLDL

**Fig 3:**
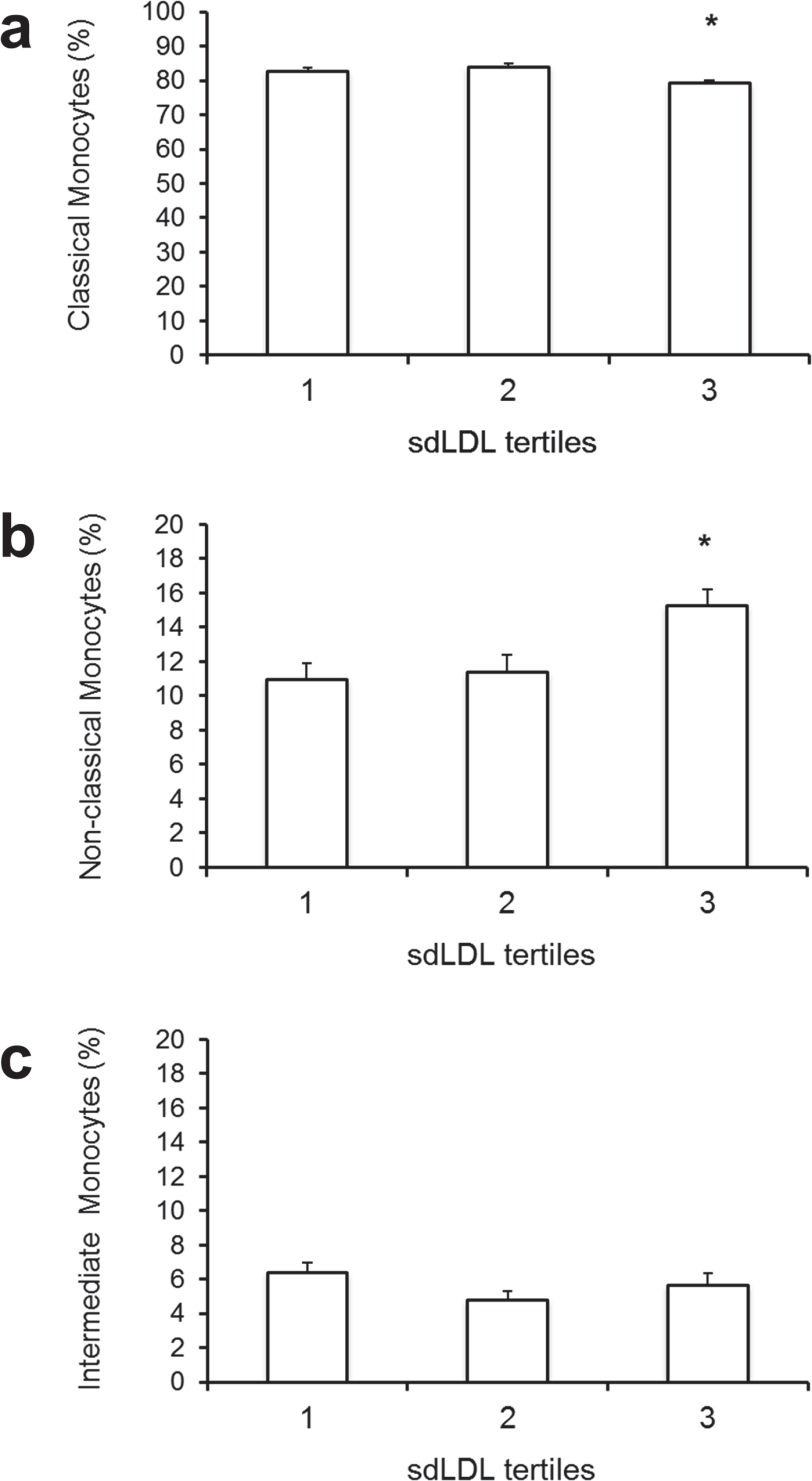
Monocyte subset distribution according to sdLDL tertiles. Monocyte subset distribution is associated with the small dense LDL subfraction. Bar graphs indicate mean % of total monocytes and error bars represent the standard error of the mean. n = 90; * p<0.01 for the lower tertiles of sdLDL vs. the third tertile

Although sdLDL only demonstrated a moderate correlation with the proportion of circulating NCM in the total study population, patients with sdLDL levels in the highest tertile showed strikingly increased levels of the pro-inflammatory NCM when compared to patients in the middle and lowest tertile. (15.2±7% vs. 11.4±6% and 10.9±4%, respectively; p<0.01 for tertile 3 vs. tertile 1 and tertile 2; Fig 3). Furthermore, patients in the highest sdLDL tertile showed also significantly lower proportion of CM than patients in the middle and lowest tertile (79.2±8% vs. 83.9±7% and 82.7±5%; p<0.01 for T3 vs. T2+T1). IM were not related to sdLDL tertiles (5.6±4% vs. 4.6±3% vs. 6.4±3% for T3, T2 and T1, respectively). Regression analysis revealed that the highest tertile of sdLDL was associated with CM independently of age, sex and BMI (β = -0.28; p = 0.01), statin dose (β = -0.26; p = 0.015) and hsCRP levels (β = -0.27; p = 0.011), respectively. The highest tertile of sdLDL was also independently associated with circulating NCM after adjustment for age, sex and BMI (β = 0.31; p = 0.004), statin dose (β = 0.30; p = 0.004), and hsCRP levels (β = 0.31; p = 0.004), respectively. In contrast to patients with sdLDL in the third tertile, patients with total LDL in the third tertile (>100 mg/dL) showed only a trend to a decreased proportion of CM (80.2±7.8 vs. 82.5±6.5 and 83.7±5.5%; p = 0.058) and no significant differences in NCM (13.3±5.8 vs. 12.3±6.8 and 11.1±4.8%; p = 0.16) or IM (6.3±3.6 vs. 5.2±2.4 and 5.3±3.8%; p = 0.96) compared to patients with LDL in the lower tertiles.

### Association of circulating inflammatory markers and tertiles of sdLDL

**Fig 4:**
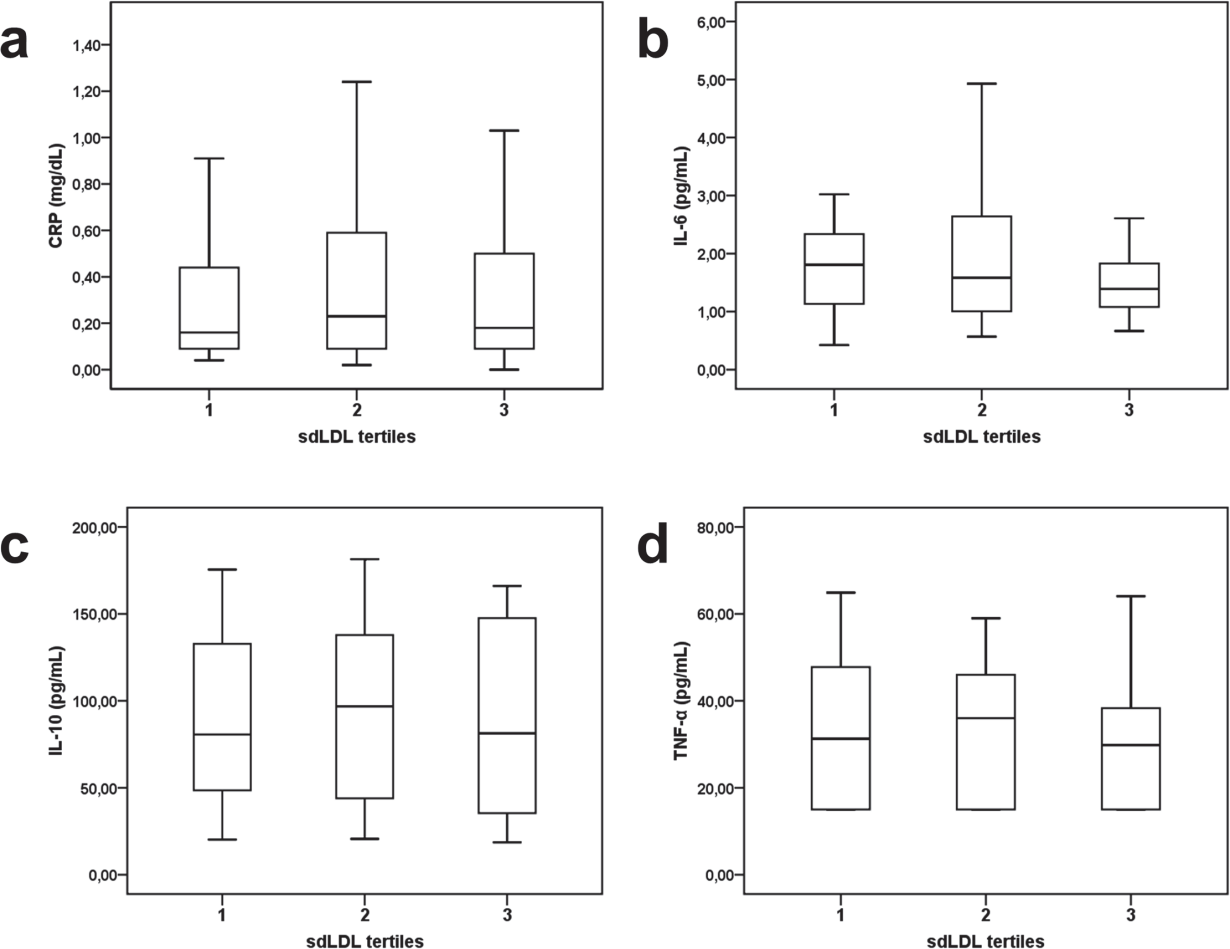
Association of circulating inflammatory cytokines and tertiles of sdLDL. Plasma levels of C-reactive protein, interleukin-6, interleukin-10 and tumor necrosis factor-α according to tertiles of small dense LDL are given. Box plots represent median and interquartile range (range from the 25^th^ to the 75^th^ percentile).

The median level of high sensitivity C-reactive protein (hsCRP) in the total patient cohort was 0.2 (IQR: 0.09–0.5) mg/dL and did not differ according to sdLDL tertiles (p = 0.76; Fig 4A). Additionally, neither plasma levels of two other pro-inflammatory markers, interleukin-6 (IL-6) (p = 0.30; Fig 4B) and tumor necrosis factor- α (TNF-α) (p = 0.88; Fig 4C) showed an association to sdLDL tertiles, nor the anti-inflammatory cytokine IL-10 (p = 0.86; Fig 3D). Furthermore, besides a moderate correlation between hsCRP-levels and intermediate monocytes (ρ = 0.24, p<0.05), there were no associations between inflammatory markers and monocyte subsets to be found (data not shown).

## Discussion

In the present study, we provide evidence for the first time that in patients with stable coronary artery disease and high levels of pro-atherogenic small dense LDL particles, monocyte subset distribution is skewed to a more "pro-inflammatory" profile with elevated levels of non-classical monocytes (CD14+CD16++) and reduced levels of classical monocytes (CD14++CD16-). This association was independent of BMI, statin dose and hsCRP-levels. The small proportion of intermediate monocytes (CD14++CD16+) did not differ according to sdLDL tertiles.

Monocytes and monocyte-derived macrophages have been implicated in all stages of atherogenesis, from initiation and progression, to destabilization and rupture of atherosclerotic lesions with possible detrimental outcome.[24–26] Monocyte heterogeneity was established by Passlick et al by staining cells with the LPS co-receptor CD14 and the FcγIII receptor CD16.[3] The vast majority of cells (approx. 85–90%) did not stain for CD16 and were termed “classical monocytes”, while the CD16+ subclass was named “non-classical monocytes”. The latter population was soon considered as pro-inflammatory, as these cells responded with a stronger production of inflammatory cytokines such as TNF-α upon activation and were shown to be proportionally elevated in diseases with underlying inflammation such as sepsis, tuberculosis and HIV infection.[6, 27] In a study including both patients with stable CAD and acute coronary syndrome, monocyte subset distribution was skewed to an increased proportion of CD16-positive cells when compared with healthy controls (13.6% versus 11.4%, respectively).[8] In over 600 apparently healthy adults, CD16+ monocytes correlated with intima media thickness.[9] Furthermore, CD16-positive cells were associated with coronary fibrous cap thickness in patients with unstable angina and with signs of plaque vulnerability as assessed by computer tomography in a cohort of patients with stable CAD.[28, 29] Additional studies evaluated monocyte subset distribution as a potential prognosis marker with conflicting results. In patients with chronic kidney disease, the CD14++CD16+ subset independently predicted cardiovascular outcome, while the classical subset was proven to be predictive for cardiovascular events in a general population. However, the latter study exhibits technical limitations, as cells were frozen for over 20 years prior to analysis.[30–32] The largest study so far evaluating monocyte subset distribution as an outcome predictor in more than 900 stable CAD patients established the intermediate monocyte population as an independent predictor of cardiovascular events.[13]

Hypercholesterolemia is considered a major risk factor for the development of atherosclerosis. As a response to the accumulation and modification of LDL within the vessel wall, monocytes migrate into the intima taking up modified LDL-particles thereby initiating plaque growth. Subset-specific interaction with lipoprotein metabolism has been suggested by several in vitro and in vivo studies, indicating specific expression of scavenger receptors and binding of oxidized and enzymatically modified lipoproteins.[33–35] In a small cross-sectional study of hypercholesterolemic patients, HDL levels were inversely correlated with non-classical monocytes, while other subpopulations were not related to lipoprotein plasma levels.[11] In another study of the same group evaluating a bigger group of hypercholesterolemic patients (n = 79), the proportion of non-classical monocytes was associated with total cholesterol, triglycerides and LDL-cholesterol, the latter one showing only a non-significant weak correlation. Interestingly, in contrast to their first study, HDL-cholesterol did not correlate with NCM.[12]

In our study, including only patients with angiographically proven stable CAD, we could show a statistically significant inverse correlation between percentage levels of CM and both total cholesterol and LDL, while NCM did not correlate with either LDL, HDL or total cholesterol, which is in contrast to the above described findings in the literature. Important discrepancies between the populations studied might explain these diverging findings, as in our population about 85% of patients were treated with a statin, in contrast to the two populations discussed above.[11] Furthermore, we described for the first time a correlation of IM with LDL-cholesterol.

Despite the obvious link between hypercholesterolemia and atherogenesis, many individuals with LDL-levels within the normal range, still develop atherosclerotic disease.[36] This suggests a significant heterogeneity of LDL-particles, as the subfraction of small dense LDL supposedly exhibits enhanced atherogenic potential.[17] Proposed mechanisms include a stronger predisposition for oxidation, lower LDL-receptor affinity and an increased accumulation within the vascular wall.[16, 37, 38] Several cross-sectional and prospective studies have suggested an association of elevated sdLDL levels with the presence of cardiovascular disease.[18–20, 39] Recently, it has been shown within the ARIC-study population consisting of 11419 men and women that sdLDL plasma levels were associated with incident coronary heart disease in a model including established risk factors.[40]

In our study, sdLDL levels showed a weak correlation with NCM. However, when patients were stratified according to tertiles of sdLDL levels, patients in the highest sdLDL tertile show a more pro-inflammatory distribution of monocyte subsets. Additionally, CM levels were lowest in patients in within the highest sdLDL tertile. These findings were independent of BMI, statin dose and hsCRP levels. The latter findings are of importance, as in another study including 166 overweight patients, the correlation between LDL and monocyte subsets was diminished after adjusting for BMI, which remained the only significant regressor for monocyte subset distribution, as detected by multivariate regression analysis.[10] In the I LIKE HOMe study, including 622 apparently healthy volunteers not receiving lipid-lowering therapy, a positive correlation between plasma triglycerides and NCM was demonstrated, as well as a weak negative correlation between plasma HDL and NCM. Again, adjustment for BMI eliminated these correlations.[9] In our study, BMI did not differ between patients according to their sdLDL tertiles and did not influence the association between monocyte subset distribution and sdLDL plasma levels. Several studies have reported conflicting results regarding the effects of statin therapy on monocyte subset distribution.[12, 41, 42] A small observational study in patients after heart transplantation demonstrated that statins depleted both circulating classical and non-classical monocytes. Patients receiving atorvastatin showed a stronger reduction in CM as compared to patients receiving pravastatin, who exhibited a strong decrease in NCM.[43] Another very small study including patients on chronic hemodialysis, demonstrated that simvastatin treatment reduced CD14 expression on circulating human monocytes.[44] Temporal cessation of statin treatment for two weeks in 66 stable CAD patients could not demonstrate an effect on circulating monocyte subset numbers, as demonstrated recently.[42] In a study including approximately 80 hypercholesterolemic patients, fluvastatin treatment combined with diet was compared with diet alone in its effects on monocyte subset distribution. Interestingly, fluvastatin treatment for 1 year lead to a 25% increase of CM as compared to a decrease of 75% of NCM.[12] This is a surprising outcome of a study examining only two subsets of monocytes in patients taking a statin not commonly used anymore; in our study, only 1 patient was treated with fluvastatin. In hypercholesterolemic patients, exercise training on top of rosuvastatin treatment lead to a small, but significant decrease in the proportion of inflammatory monocytes.[41]

Here we could demonstrate that monocyte subsets were not associated with statin treatment and that the association between sdLDL and monocyte subset distribution was independent of statin treatment dose.

In addition, we demonstrated that sdLDL plasma levels exhibit no association with circulating pro- and anti-inflammatory markers, namely hsCRP, IL-6, IL-10 and TNF-α. This is in line with previously published literature, as in the Quebec Cardiovascular Study cohort including 2025 men free of CAD at baseline, sdLDL levels only marginally correlated with markers of inflammation such as hsCRP.[45] In the literature, non-classical monocytes were defined as a major source of TNF.[6] In a study comparing patients with coronary artery disease and apparently disease-free subjects, though mixing acute MI patients and stable CAD patients, TNF-α showed a correlation with non-classical monocytes in a model with only two monocyte subsets.[6, 8] Severely injured patients showed a correlation between CRP levels and the intermediate subset, a possible cellular hallmark of acute illness.[46] In patients with acute erysipelas, CRP and IL-6 levels correlated with an inflammatory monocyte subset. However, these cells exhibited reduced intracellular TNF protein as compared to classical monocytes.[47] This highlights the complex and far from fully understood mechanisms of inflammatory cytokine production in monocyte subsets during inflammatory activation. Interestingly, besides a weak correlation between hsCRP and intermediate monocytes, no correlations between circulating inflammatory markers and monocyte subsets were shown in our study population. Therefore one may speculate that elevated plasma levels of sdLDL exert some of its detrimental effects via modulation of monocyte subset distribution to a rather pro-inflammatory and pro-atherogenic profile, rather than directly influencing classical inflammatory pathways.

Some limitations of the present study have to be acknowledged. First, this is a single center study with a rather small number of patients. Furthermore, the cross-sectional study design only allows us to outline associations between monocyte subset distribution and LDL subfractions, while we cannot draw functional insights into monocyte subset plasticity in atherosclerotic disease. In addition, we did not include a control group with absence of coronary stenosis at coronary angiography. This would be of particular interest, as the absence of low-grade vascular inflammation would help to assess possible direct effects of sdLDL on monocytes. However, our results indicate a link between innate immunity and lipid metabolism in stable atherosclerosis.

In conclusion, this study provides evidence for the first time for an association between plasma levels of atherogenic sdLDL particles and an increased proportion of non-classical monocytes and a smaller classical monocyte population. These results might represent a new link between an atherogenic lipoprotein phenotype and innate immunity in stable atherosclerotic disease. Further studies are required to gain functional insight into the mechanistic relationship between monocyte subsets and small dense LDL and their specific roles in atherogenesis.
